# Identification of Chloride Channels CLCN3 and CLCN5 Mediating the Excitatory Cl^−^ Currents Activated by Sphingosine-1-Phosphate in Sensory Neurons

**DOI:** 10.3389/fnmol.2018.00033

**Published:** 2018-02-09

**Authors:** Yanmei Qi, Norbert Mair, Kai K. Kummer, Michael G. Leitner, María Camprubí-Robles, Michiel Langeslag, Michaela Kress

**Affiliations:** ^1^Division of Physiology, Department of Physiology and Medical Physics, Medical University of Innsbruck, Innsbruck, Austria; ^2^Department of Neurophysiology, Philipps University of Marburg, Marburg, Germany

**Keywords:** CLCN3, CLCN5, DRG neurons, Rho, Sphingosine 1-phosphate, TRPV1

## Abstract

Sphingosine-1-phosphate (S1P) is a bioactive sphingolipid involved in numerous physiological and pathophysiological processes. We have previously reported a S1P-induced nocifensive response in mice by excitation of sensory neurons via activation of an excitatory chloride current. The underlying molecular mechanism for the S1P-induced chloride conductance remains elusive. In the present study, we identified two CLCN voltage-gated chloride channels, CLCN3 and CLCN5, which mediated a S1P-induced excitatory Cl^−^ current in sensory neurons by combining RNA-seq, adenovirus-based gene silencing and whole-cell electrophysiological voltage-clamp recordings. Downregulation of CLCN3 and CLCN5 channels by adenovirus-mediated delivery of shRNA dramatically reduced S1P-induced Cl^−^ current and membrane depolarization in sensory neurons. The mechanism of S1P-induced activation of the chloride current involved Rho GTPase but not Rho-associated protein kinase. Although S1P-induced potentiation of TRPV1-mediated ionic currents also involved Rho-dependent process, the lack of correlation of the S1P-activated Cl^−^ current and the potentiation of TRPV1 by S1P suggests that CLCN3 and CLCN5 are necessary components for S1P-induced excitatory Cl^−^ currents but not for the amplification of TRPV1-mediated currents in sensory neurons. This study provides a novel mechanistic insight into the importance of bioactive sphingolipids in nociception.

## Introduction

Sphingosine-1-phosphate (S1P) is a biologically active sphingolipid that is involved in numerous cellular functions, such as cell migration and morphogenesis (Kupperman et al., 2000; Pyne and Pyne, 2000), lymphocyte egress (Pappu et al., 2007), angiogenesis and neurogenesis (Kono et al., 2004; Mizugishi et al., 2005). S1P is generated through conversion of ceramide into sphingosine by means of ceramidases and subsequent phosphorylation of sphingosine by sphingosine kinases (Pyne and Pyne, 2000; Spiegel and Milstien, 2003). Human platelets contain high concentrations of sphingolipids, particularly S1P, which is actively released into circulation upon platelet activation by physiological agonists (e.g., thrombin and collagen) or by damage to blood vessel such as surgery or local trauma (Yatomi et al., 1995, 1997; Tani et al., 2005; Golebiewska and Poole, 2015; Vito et al., 2016), resulting in micromolar S1P concentrations in blood plasma (Murata et al., 2000; Schmidt et al., 2006; Ohkawa et al., 2008; Ono et al., 2013). In general, S1P exerts its pleiotropic effects by signaling through a family of S1P receptors, consisting of five G-protein-coupled receptors designated S1PR_1–5_ (Spiegel and Milstien, 2003; Salvemini et al., 2013). S1P receptors are expressed in a wide variety of tissues including brain, spinal cord and dorsal root ganglia (DRG). S1PR_1_, S1PR_4_ and S1PR_5_ subtypes are mainly coupled to G_αi_, whereas S1PR_2_ and S1PR_3_ subtypes are coupled to G_αi_, G_q_ and G_α12/13_ (Spiegel and Milstien, 2003; Brinkmann, 2007).

Pain is an unpleasant sensory and emotional experience associated with tissue damage, and millions of individuals suffer from acute or chronic pain every year. It has been well established that the perception of pain is initiated by the activation of peripheral sensory afferents and hypersensitization of peripheral sensory neurons contributes to the development of inflammatory and neuropathic pain (Khan et al., 2002; Shim et al., 2005; Xiao and Bennett, 2007; Devor, 2009; Berta et al., 2017). Previous studies have suggested the importance of the bioactive lipid S1P in peripheral sensitization (Joseph and Levine, 2004; Zhang et al., 2006a,b; Doyle et al., 2011; Mair et al., 2011; Welch et al., 2012; Camprubí-Robles et al., 2013; Salvemini et al., 2013; Langeslag et al., 2014; Li et al., 2015). For example, S1P/S1PR_1_ signaling enhances the activity of TRPV1 channel (Langeslag et al., 2014), a vital ion channel in nociceptors for heat transduction and pain sensitization (Li et al., 2008; Wang, 2008; Basso and Altier, 2017; Berta et al., 2017), resulting in enhanced thermal hypersensitivity in mice (Mair et al., 2011). Apart from that, S1P mediates nerve growth factor (NGF)- and TNF-α-induced sensitization of sensory neurons (Joseph and Levine, 2004; Zhang et al., 2006b; Doyle et al., 2011), implying that S1P may regulate neuronal excitability. Moreover, we and others have shown that S1P in preclinical models enhanced the excitability of sensory neurons *in vitro* and elicited spontaneous pain behavior *in vivo* as well as signatures of nociceptor activation in humans (Zhang et al., 2006a; Mair et al., 2011; Camprubí-Robles et al., 2013; Li et al., 2015), suggesting that peripherally released S1P may evoke significant nociception by directly exciting peripheral neurons.

Furthermore, we have demonstrated that S1P excites DRG neurons via evoking an excitatory ionic current by activation of a chloride conductance (Camprubí-Robles et al., 2013). A similar depolarizing chloride current elicited by S1P has been reported in other cell types such as N1E-115 neuroblastoma cells (Postma et al., 1996, 2001). To date, the underlying molecular basis for the S1P-evoked chloride conductance remains to be elucidated. In the present study we aimed to identify the chloride channel that mediates the S1P-activated chloride conductance in sensory neurons.

## Materials and Methods

### Ethics Statement

All animal breeding and experiments have been performed with permission of the Austrian BMWF ministry (BMWF-66.011/0113-II/3b/2010; BMWF-66.011/0051-II/10b2008; GZ 66.011/85-C/GT/2007) and according to ethical guidelines of the IASP (International Association for the Study of Pain).

### DRG Neurons Culture

Lumbar (L1–L6) DRG containing the cell bodies of primary afferents that project into the hindpaw were harvested from adult C57BL/6J mice (age 8–12 weeks) as previously published (Camprubí-Robles et al., 2013; Langeslag et al., 2014). Briefly, ganglia were treated enzymatically with Liberase Blendzyme 1 (9 mg/100 ml DMEM, Roche) for two times 30 min and 1× Trypsin-EDTA (Invitrogen) for 15 min. The DRG were then washed and dissociated mechanically in serum-free TNB^®^ medium (Biochrom) with a fire-polished Pasteur pipette, and centrifuged through a 3.5% BSA gradient (Sigma) to eliminate non-neuronal cells and debris. The resulting sensory neurons were resuspended, plated on poly-L-lysine/laminin coated coverslips and cultivated in TNB medium supplemented with NGF (25 ng/ml), L-glutamine, penicillin G sodium and streptomycin sulfate (all from Invitrogen) at 37°C in 5% CO_2_ for 24 h, unless otherwise indicated.

### Adenoviral shRNA Infection of DRG Neurons

The shRNA adenoviruses Ad-GFP-U6-m-CLCN3-shRNA (shADV-255571), Ad-GFP-U6-m-CLCN4-shRNA (shADV-255572) and Ad-GFP-U6-m-CLCN5-shRNA (shADV-255574) were purchased from Vector Biolabs. A non-specific scrambled shRNA adenovirus Ad-GFP-U6-scrambled-shRNA (Vector Biolabs, 1122N) expressing green fluorescent protein (GFP) alone was used as a control.

For adenoviral infection, after centrifugation of dissociated DRG in 3.5% BSA gradient, the resulting pellet was resuspended in serum-free TNB medium containing Ad-GFP-U6-scrambled-shRNA, Ad-GFP-U6-m-CLCN3-shRNA, Ad-GFP-U6-m-CLCN4-shRNA or Ad-GFP-U6-m-CLCN5-shRNA at a concentration of 2 × 10^8^ pfu/mL. The mixture was then plated on coverslips or 24-well culture dishes (Nunc) coated with poly-L-lysine/laminin and incubated at 37°C in 5% CO_2_. Two hours after incubation, the medium was replaced with fresh supplemented TNB medium. Three days after infection, adenovirus-infected neurons were used for electrophysiological recording and RNA extraction. The shRNA adenoviral vector contained a reporter gene encoding GFP under the control of U6 promoter, and all electrophysiological recordings were made from GFP-positive neurons only.

### Electrophysiology

Whole-cell patch-clamp recordings were performed using patch pipettes with a tip resistance of 2–4 MΩ as previously described (Camprubí-Robles et al., 2013). Ionic currents were recorded from isolated DRG neurons in the whole-cell voltage-clamp configuration at −60 mV holding potential, unless otherwise indicated. S1P-induced currents and capsaicin-induced currents were measured from baseline to peak. The external solution (ECS) contained (in mM): 145 NaCl, 5 KCl, 2 CaCl_2_, 1 MgCl_2_ (all Sigma), 10 glucose and 10 HEPES (Merck, Darmstadt, Germany), at pH 7.3 adjusted with NaOH (Merck), and electrodes were filled with internal solution (ICS, in mM): 140 KCl, 2 MgCl_2_, 2 Na-ATP, 0.2 Na-GTP, 0.1 CaCl_2_, 1 EGTA (all Sigma) and 10 HEPES (Merck), at pH 7.3 adjusted with KOH (Merck). For the experiments recorded after treatment with C3 toxin and Y-27632, low Ca^2+^ ECS was used, containing 145 NaCl, 5 KCl, 0.1 CaCl_2_, 1 MgCl_2_ (all Sigma), 10 glucose and 10 HEPES (Merck, Darmstadt, Germany), at pH 7.3 adjusted with NaOH (Merck). The membrane potential was recorded using current-clamp configuration using an ECS containing the following (in mM): 145 NaCl, 5 KCl, 2 CaCl_2_, 1 MgCl_2_ (all Sigma), 10 D-glucose and 10 HEPES (Merck), at pH 7.3 adjusted with NaOH (Merck). The pipette solution was composed (in mM) of 45 KCl, 98 K-gluconate, 0.5 CaCl_2_, 5 EGTA, 10 HEPES, 2 MgATP, 0.2 NaGTP, pH 7.3 adjusted with KOH (Merck).

A seven-barrel system with common outlet was used for fast drug administration (WAS 02, Dittel, Prague). Neurons were visualized with an inverted microscope (Zeiss, Germany) equipped with a CCD camera and MetaFluor^®^ fluorescence imaging software (Molecular Devices). Membrane current and voltage were filtered at 2.9 kHz, sampled at 1 kHz and recorded with an EPC-10 amplifier (HEKA, Germany) and the Patchmaster software (HEKA). Acquired traces were analyzed using Patchmaster and Fitmaster software (HEKA). Pipette and membrane capacitance were compensated using the auto function of Patchmaster. Voltage-gated currents were evoked using a standard series of voltage commands. Briefly, the neurons were depolarized from −60 to +40 mV in increments of 5 mV (40 ms test pulse duration). All experiments were performed at room temperature. Sphingosine-1-phosphate (S1P) and Capsaicin were purchased from Sigma Aldrich. Cell-permeable C3 toxin was from Cytoskeleton (Tebu-bio), and Y-27632 was from Calbiochem.

### RT-PCR

Total RNA was isolated from murine DRG explants and primary cultures of DRG neurons by using peqGOLD TriFast (PeqLab) as previously described (Malsch et al., 2014). The quantity of RNA was analyzed using Nanodrop 2000 (ThermoScientific). Total RNA was reverse transcribed using MuLv reverse transcriptase (2.5 U/μl, Applied Biosystems) with random hexamer primers (10 ng/μl), RiboLock (2 U/μl), 1× Taq Buffer (all from Thermo Scientific), MgCl_2_ (5 mM) and dNTPs (1 mM, Fermentas), followed by PCR performed with gene specific primers. The primer sequences used in this study were listed in Table 1. Mouse β-actin was used as an internal standard. The thermal cycling protocol was 94°C for 30 s, 55°C for 30 s and 72°C for 30 s. All PCR reactions were cycled 30 times. The amplified PCR products were visualized following electrophoresis in 1% agarose gels containing SYBR Safe stain (Thermo Fisher Scientific).

**Table 1:** Primer pairs used for PCR amplifications.

Gene	GenBank Accession No.	Primer sequence (5′-3′)	Length (bp)
*Clcn2*	NM_009900	Forward: TGAGTCCATGATCCTACTG	309
		Reverse: CCTGCTGACTCCATGTTG	
*Clcn3*	NM_007711	Forward: CCTCTTTCCAAAGTATAGCAC	549
		Reverse: CTGGCATTCATGTCATTTC	
*Clcn4*	NM_011334	Forward: GAGGACTTCCACACCATA	411
		Reverse: TGCAAACAGCAACGCCCATA	
*Clcn5*	NM_016691	Forward: GGAACATCTTGTGCCACTG	563
		Reverse: TGTGTTGAAGTGGTTCTC	
*Clcn6*	NM_011929	Forward: TCTTCCACGAGTCAAACC	406
		Reverse: TCATCCTTACAACCCCAC	
*Clcn7*	NM_011930	Forward: GCTCCTGCCTTTCAGTTGTC	219
		Reverse: TTCAAGAACTGCACCACTGC	

### Microfluorimetric Calcium Measurements

Calcium imaging was performed as previously described (Camprubí-Robles et al., 2013). Briefly, cultured cells were non-disruptively loaded with 3 μM of the Ca^2+^ sensitive dye Fura-2 AM (Invitrogen) in ECS containing (in mM): 145 NaCl, 5 KCl, 2 CaCl_2_, 1 MgCl_2_, 10 D-glucose (all from Sigma) and 10 HEPES (Merck), at pH 7.3 adjusted with NaOH (Merck) and were incubated at 37°C in 5% CO_2_ for 25 min. Then cells were washed and kept in ECS for experiments. Experiments were performed using an Olympus IX71 microscope (Olympus) with a 20×/0.85 N.A. oil-immersion objective (Olympus). Fura-2 was excited at 340 nm and 380 nm (excitation time: 25 ms) with a polychrome IV monochromator (TILL Photonics), and fluorescence intensities were filtered by a 510 nm LP filter and recorded with a CCD camera (CoolSNAP ES, Photometrics). The ratio of fluorescence intensities exited at 340 nm and 380 nm (F340/380) was calculated after background correction, and the changes of intracellular Ca^2+^ signal were depicted as ratio change (∆F340/380) measured as peak F340/380 ratio over baseline. For data acquisition, MetaFluor4.6r8 (Molecular Devices) was used and off-line analysis was performed with OriginPro7.SR2 (Origin Lab). The threshold for S1P-positive cells was set to fourfold the SD of the Ca^2+^ signal evoked by 0.1% methanol (∆F340/380 = 0.04).

### Data and Statistical Analysis

All statistical comparisons were two-sided and were performed with Graphpad Prism 7 software. For all *in vitro* experiments, recordings were pooled from at least three mice. Unpaired *t*-test, Mann-Whitney *U* test, Fisher’s exact test and two-way ANOVA analysis were used for two-group comparison. The association between S1P-induced Cl^−^ current and S1P-induced potentiation of I_caps_ was tested by Pearson correlation coefficient calculation. Differences with a *p* < 0.05 were considered to be statistically significant. All results are expressed as mean ± standard error of the mean (SEM).

## Results

### Expression of Voltage-Gated Chloride Channels in Sensory Neurons

**Figure 1:**
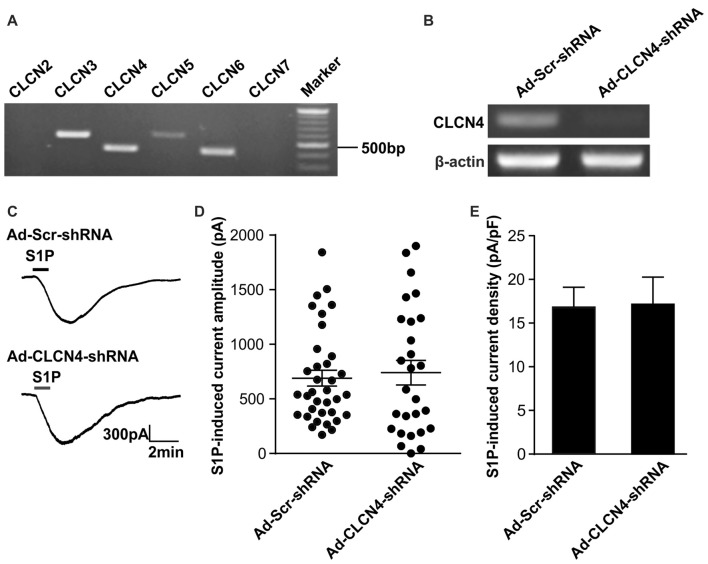
Down-regulation of *Clcn4* mRNA does not alter S1P-induced Cl^−^ current in sensory neurons. **(A)** Expression of chloride channels in mouse dorsal root ganglia (DRG). Representative RT-PCR results for *Clcn2*, *Clcn3*, *Clcn4*, *Clcn5*, *Clcn6* and *Clcn7* mRNA in intact DRG. Marker: 100bp DNA ladder. **(B)** Cultured DRG neurons were infected with scrambled shRNA adenovirus (Ad-Scr-shRNA) or adenovirus encoding specific shRNA against CLCN4 (Ad-CLCN4-shRNA). Representative RT-PCR results from three independent experiments showed that *Clcn4* mRNA level was reduced 3 days after infection by CLCN4 shRNA adenovirus but not with scrambled shRNA adenovirus. **(C)** Example traces of excitatory inward currents (carried by Cl^−^ efflux) induced by 1 μM S1P recorded in DRG neurons infected with scrambled shRNA adenovirus or CLCN4 shRNA adenovirus at −60 mV holding potential. **(D)** Dot-plot depicting the amplitude of S1P-induced inward current between Ad-Scr-shRNA and Ad-CLCN4-shRNA groups. Each dot represents a single cell. The mean values and standard error of the mean (SEM) bar were indicated in the graph. There was no difference in the amplitude of S1P-evoked inward current between Ad-Scr-shRNA and Ad-CLCN4-shRNA groups (Mann-Whitney *U* test, *n* = 27–35, *p* > 0.05). **(E)** Bar-chart showing that knockdown of CLCN4 level with adenovirus-mediated shRNA did not alter S1P-induced current density in DRG sensory neurons (Mann-Whitney *U* test, *n* = 27–35, *p* > 0.05).

Previous ionic substitution and pharmacological inhibition experiments have revealed that S1P-induced Cl^−^ current is mediated by Ca^2+^-independent Cl^−^ channels (Camprubí-Robles et al., 2013). To identify the S1P-activated chloride channel in DRG neurons, we thus first searched for Ca^2+^-independent Cl^−^ channels that are expressed in DRG based on our RNA sequencing data from wild-type mouse DRG explants. Several CLCN channels of voltage**-**gated chloride channel family were detected by RNA sequencing, spanning from CLCN2–CLCN7 (Table 2). RT-PCR analysis validated *Clcn3*, *Clcn4*, *Clcn5* and *Clcn6* mRNA expression in both DRG explants and cultured DRG neurons (Figure 1A and Supplementary Figures S1, S4), while there was lack of expression of *Clcn2* and *Clcn7* mRNA in both sample types (Figure 1A and Supplementary Figure S1).

**Table 2:** List of chloride channels detected by RNA sequencing from wild-type mouse dorsal root ganglia (DRG) explants.

Gene symbol	Gene name	RPKM value
Clcn2	Voltage-gated chloride channel 2	5.7
Clcn3	Voltage-gated chloride channel 3	25.7
Clcn4	Voltage-gated chloride channel 4	31.0
Clcn5	Voltage-gated chloride channel 5	6.9
Clcn6	Voltage-gated chloride channel 6	26.0
Clcn7	Voltage-gated chloride channel 7	11.7
Clic1	Chloride intracellular channel 1	69.9
Clns1a	Chloride channel, nucleotide-sensitive, 1A	11.2

*RPKM: Reads per kilo base per million mapped reads*.

### Chloride Channels CLCN3 and CLCN5 Mediate S1P-Induced Excitatory Conductance in Sensory Neurons

The four identified chloride channels (CLCN3–6) reside predominantly in intracellular membranes of the endocytotic-lysosomal pathway (Jentsch et al., 2005a,b). However, CLCN3 and CLCN4, together with CLCN5, have also been suggested to act as Cl^−^ channels at the plasma membrane (Kawasaki et al., 1995; Friedrich et al., 1999; Huang et al., 2001; Jentsch et al., 2005a). Therefore we selected CLCN3, CLCN4 and CLCN5 as possible candidates and investigated whether these three chloride channels contribute to S1P-activated chloride conductance in sensory neurons. The expression of each chloride channel candidate was down-regulated using an adenovirus-based RNAi knockdown strategy in DRG neurons and 72 h post-infection the S1P-activated currents were recorded using the whole-cell voltage clamp configuration of the patch-clamp technique.

**Figure 2:**
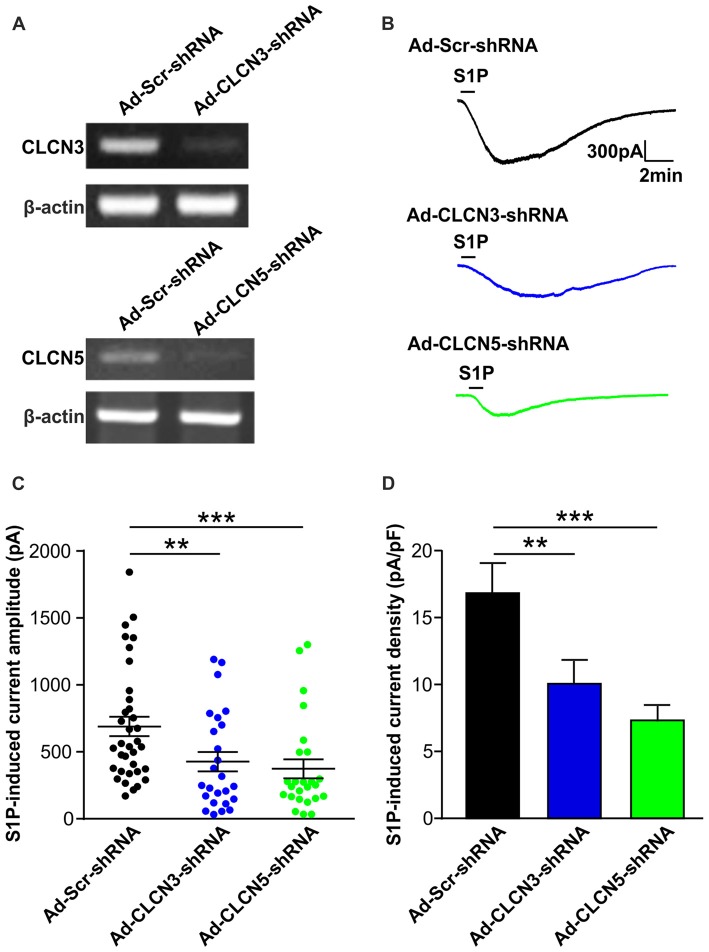
Adenovirus-mediated underexpression of CLCN3 or CLCN5 by shRNA reduces S1P-induced Cl^−^ current in mouse sensory neurons. **(A)** Cultured DRG neurons were infected with scrambled shRNA adenovirus (Ad-GFP-U6-scrambled shRNA) or adenovirus carrying specific shRNA against CLCN3 (Ad-GFP-U6-CLCN3 shRNA) or CLCN5 (Ad-GFP-U6-CLCN5 shRNA). Representative RT-PCR results from three independent experiments showed that *Clcn3* and Clcn5 mRNA levels were reduced by their corresponding specific shRNA 3 days after adenoviral infection. **(B)** Representative whole-cell voltage clamp recording of inward Cl^−^ currents induced by S1P (1 μM, 1 min) from DRG neurons infected with Ad-Scr-shRNA, Ad-CLCN3-shRNA or Ad-CLCN5-shRNA adenovirus at −60 mV holding potential. **(C)** Dot-plot graph showing the amplitude of S1P -induced inward current in Ad-Scr-shRNA (*n* = 35), Ad-CLCN3-shRNA (*n* = 25) and Ad-CLCN5-shRNA (*n* = 25) groups. Each dot represents a single cell and mean and SEM are indicated. Note the reduced amplitude of S1P-activated inward current by Ad-CLCN3-shRNA or Ad-CLCN5-shRNA in DRG neurons 3 days after viral infection (Mann-Whitney *U* test, Ad-Scr-shRNA vs. Ad-CLCN3-shRNA **p < 0.01, Ad-Scr-shRNA vs. Ad-CLCN5 shRNA ***p < 0.001). **(D)** Quantitative comparison of S1P-induced current density (current amplitude/cell capacitance, pA/pF) in DRG neurons after adenoviral infection of Ad-Scr-shRNA, Ad-CLCN3-shRNA or Ad-CLCN5-shRNA. Adenovirus-mediated knockdown of CLCN3 or CLCN5 by shRNA significantly reduced S1P-induced current density in DRG neurons (Mann-Whitney *U* test, Ad-Scr-shRNA vs. Ad-CLCN3-shRNA **p < 0.01, Ad-Scr-shRNA vs. Ad-CLCN5 shRNA ***p < 0.001).

To confirm the efficiency of adenovirus-based RNAi knockdown in DRG neurons, the mRNA level of each candidate was assessed with the RT-PCR technique also 72 h after adenoviral infection. Analysis of RT-PCR results confirmed efficient reduction of *Clcn3*, *Clcn4* and *Clcn5* mRNA levels by viral delivery of their corresponding shRNA (Figures 1B, 2A and Supplementary Figures S5, S6). In line with our previous report (Camprubí-Robles et al., 2013), application of S1P (1 μM, 1 min) evoked a slowly activating and deactivating inward current in most of the recorded neurons infected with virus carrying scrambled shRNA (Ad-Scr-shRNA), with a peak current amplitude of 689.3 ± 72.4 pA (Figures 1C,D, *n* = 35 cells). Knockdown of *Clcn4* mRNA by adenovirus-mediated CLCN4 shRNA did not alter the amplitude of S1P-induced inward current (I_S1P_; Figure 1D, Ad-CLCN4-shRNA: 740.3 ± 112.3 pA in *n* = 27 neurons, Mann-Whiney *U* test, *p* = 0.874). When the S1P-induced transmembrane current was corrected for membrane capacitance, which is shown as current density (current amplitude/cell capacitance, pA/pF), no significant difference was observed between Ad-Scr-shRNA group and Ad-CLCN4-shRNA group in DRG neurons (Figure 1E, Ad-Scr-shRNA: 16.82 ± 2.3 pA/pF in *n* = 35 neurons, Ad-CLCN4-shRNA: 14.1 ± 3.5 pA/pF in *n* = 27 neurons, Mann-Whiney *U* test, *p* = 0.429). These data suggested that CLCN4 does not contribute to the S1P-induced conductance in sensory neurons.

On the contrary, delivery of virus encoding CLCN3 shRNA (Ad-CLCN3-shRNA) significantly reduced the amplitude of I_S1P_ (Figures 2B,C, I_S1P_ amplitude: 426.9 ± 72.9 pA in Ad-CLCN3-shRNA group (*n* = 25), Mann-Whiney *U* test, *p* = 0.0054) and S1P-induced current density (Figure 2D, I_S1P_ current density: 10.07 ± 1.78 pA/pF in Ad-CLCN3-shRNA (*n* = 25), Mann-Whiney *U* test, *p* = 0.0084), suggesting the S1P-induced conductance is partially mediated by chloride channel CLCN3. Similarly, downregulation of *Clcn5* mRNA expression by adenovirus-mediated CLCN5 shRNA robustly reduced I_S1P_ amplitude (Ad-CLCN5-shRNA: 373.8 ± 70.9 pA in *n* = 25 neurons, Mann-Whiney *U* test, *p* = 0.0002) and current density (7.33 ± 1.14 pA/pF in Ad-CLCN5-shRNA (*n* = 25), Mann-Whiney *U* test, *p* < 0.0001; Figures 2A–D), revealing a significant contribution of CLCN5 to the S1P-induced conductance in sensory neurons. Taken together, these data suggest that S1P-induced current in sensory neurons is mediated by at least two chloride channels, including CLCN3 and CLCN5.

### CLCN3 and CLCN5 Regulate S1P-Induced Membrane Depolarization in Sensory Neurons

**Figure 3:**
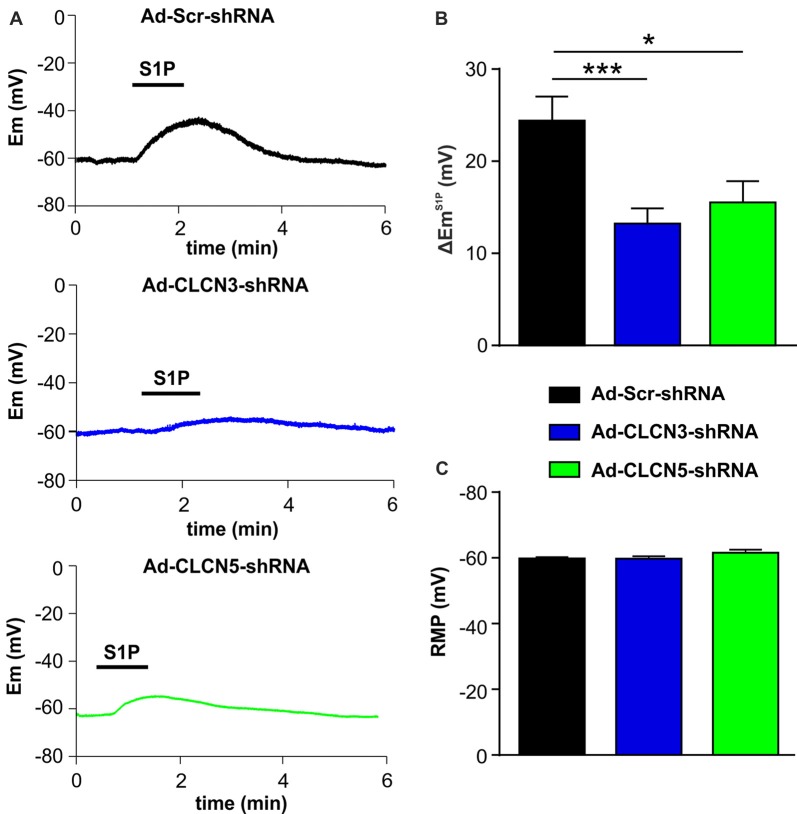
Adenovirus-based shRNA knockdown of CLCN3 or CLCN5 decreases S1P-induced membrane depolarization in sensory neurons. **(A)** Representative whole-cell current clamp recording of membrane potential evoked by stimulation of S1P (1 μM, 1 min) from DRG neurons 3 days after adenoviral infection of Ad-Scr-shRNA, Ad-CLCN3-shRNA or Ad-CLCN5-shRNA. **(B)** The depolarizing effect of S1P was significantly decreased by viral infection of Ad-CLCN3-shRNA or Ad-CLCN5-shRNA (Unpaired *t*-test, Ad-Scr-shRNA vs. Ad-CLCN3-shRNA, *n* = 29–35, ***p < 0.001, Ad-Scr-shRNA vs. Ad-CLCN5-shRNA, *n* = 32–35, *p < 0.05). **(C)** Bar-chart showing that the resting membrane potential (RMP) was not affected by viral infection of Ad-CLCN3-shRNA or Ad-CLCN5-shRNA (Unpaired *t*-test, Ad-Scr-shRNA vs. Ad-CLCN3-shRNA, *n* = 29–35, *p* > 0.05, Ad-Scr-shRNA vs. Ad-CLCN5-shRNA, *n* = 32–35, *p* > 0.05).

In a previous study, we have shown that S1P elicited a significant membrane depolarization in mouse DRG neurons, supporting a direct excitatory effect of S1P on primary afferent neurons (Camprubí-Robles et al., 2013). We therefore tested whether chloride channels CLCN3 and CLCN5 are involved in S1P-induced membrane depolarization in mouse DRG neurons. Whole-cell current clamp recordings were performed to monitor membrane potential (Em) before and during S1P exposure of adenovirus-infected DRG neurons. Application of S1P (1 μM, 1 min) resulted in a membrane potential change in Ad-scrambled shRNA-infected DRG neurons of approximately +24 mV with the Em depolarizing from −59.77 ± 0.38 mV to −35.28 ± 2.76 mV (Supplementary Figure S2, unpaired *t*-test, *n* = 29, *p* < 0.0001), confirming the depolarizing effect of S1P on DRG neurons. S1P also induced a depolarization of membrane potential in DRG neurons infected with adenovirus encoding CLCN3 shRNA or CLCN5 shRNA (Supplementary Figure S2). However, adenovirus-based shRNA knockdown of CLCN3 or CLCN5 significantly reduced the magnitude of S1P-induced Em change (∆Em^S1P^) compared with Ad-Scr-shRNA group (Figures 3A,B; Ad-Scr-shRNA: ∆Em^S1P^ = 24.42 ± 0.38 mV, *n* = 29; Ad-CLCN3-shRNA: ∆Em^S1P^ = 13.25 ± 1.39 mV, *n* = 35; Ad-CLCN5-shRNA: ∆Em^S1P^ = 15.54 ± 2.38 mV, *n* = 32; unpaired *t*-test, Ad-Scr-shRNA vs. Ad-CLCN3-shRNA *p* = 0.0004, Ad-Scr-shRNA vs. Ad-CLCN5-shRNA *p* = 0.0143). These data suggest that chloride channels CLCN3 and CLCN5 significantly contribute to the S1P-induced membrane depolarization in sensory neurons.

The membrane potential at resting state was not affected by Ad-CLCN3-shRNA or Ad-CLCN5-shRNA in DRG neurons (Figure 3C, unpaired *t*-test, Ad-Scr-shRNA vs. Ad-CLCN3-shRNA *p* = 0.9605, Ad-Scr-shRNA vs. Ad-CLCN5-shRNA *p* = 0.1077), indicating that chloride channels CLCN3 and CLCN5 most likely do not contribute to the background Cl^−^ conductance in sensory neurons. Previous reports have shown that CLCN3 and CLCN5 can be activated by large membrane depolarization when expressed in oocytes and cell lines (Kawasaki et al., 1994, 1995; Friedrich et al., 1999). We therefore examined the contribution of chloride channels CLCN3 and CLCN5 to voltage-gated currents in DRG neurons. Voltage-gated currents were recorded under whole-cell voltage clamp conditions in DRG neurons infected with adenovirus carrying scrambled shRNA, CLCN3 shRNA or CLCN5 shRNA. Current–voltage (I-V) plots showed that knockdown of CLCN3 or CLCN5 in DRG neurons by shRNA did not significantly alter voltage-gated inward currents (Supplementary Figure S3, Two-way repeated measures ANOVA, Ad-Scr-shRNA vs. Ad-CLCN3-shRNA *p* = 0.3608, Ad-Scr-shRNA vs. Ad-CLCN5-shRNA *p* = 0.7435) and outward currents (Supplementary Figure S3, Two-way repeated measures ANOVA, Ad-Scr-shRNA vs. Ad-CLCN3-shRNA *p* = 0.9225, Ad-Scr-shRNA vs. Ad-CLCN5-shRNA *p* = 0.4906), suggesting CLCN3 and CLCN5 are not significantly involved in voltage-gated currents in sensory neurons in a physiological range.

### Rho-Dependent Activation of S1P-Induced Chloride Conductance in Sensory Neurons

**Figure 4:**
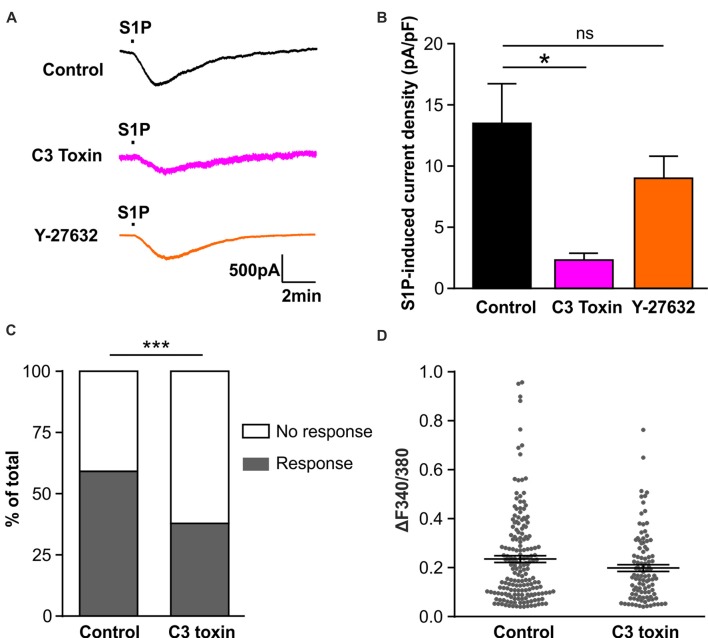
Activation of Rho but not Rho-associated protein kinase (ROCK) is required for S1P-induced activation of a Cl^−^ conductance in sensory neurons. **(A)** Example traces of inward current induced by 1 μM S1P recorded under whole-cell voltage clamp conditions in cultured DRG neurons pretreated without (control) or with Rho inhibitor C3 toxin (0.5 μg/ml, overnight) or ROCK inhibitor Y-27632 (10 μM, 30 min) at −80 mV holding potential. **(B)** Quantitative comparison of S1P-induced current density in control and drug-pretreated DRG neurons. Pretreatment with C3 toxin (0.5 μg/ml, overnight) significantly reduced S1P-induced inward current in cultured sensory neurons (Unpaired *t*-test, *n* = 13–20, *p < 0.05). No significant difference was obtained between control and Y-27632-pretreated group (Unpaired *t*-test, *n* = 14–20, *p* > 0.05). **(C)** Calcium imaging was used to record S1P-evoked Ca^2+^ transients in control and C3 toxin-pretreated DRG neurons. Stacked histograms showing that pretreatment with Rho inhibitor C3 toxin (0.5 μg/ml) significantly decreased the percentage of S1P-responding neurons (Fisher’s exact test, *n* = 273–306, ***p < 0.001). **(D)** The dot-plot graph showing no significant difference in S1P-evoked Ca^2+^ increase magnitude between control and C3-toxin treated groups (Mann-Whitney U test, *n* = 103–180, *p* > 0.05). Each dot represents a single S1P-responding cell.

We have previously reported that S1P activated the chloride conductance in mouse DRG neurons through the G-protein coupled S1PR_3_ receptor (Camprubí-Robles et al., 2013), strongly indicating that S1P did not directly activate chloride channels, but through second messengers activated upon receptor binding. Thus we set out to explore the downstream signaling events for the activation of I_S1P_ in sensory neurons. Previous studies in N1E-115 neuroblastoma cells have demonstrated the requirement of RhoA for the activation of G_α13_-mediated chloride conductance by bioactive lipids such as lysophosphatidic acid (LPA) and S1P (Postma et al., 2001; Ponsioen et al., 2009), and we recently found S1P induced the activation of RhoA in sensory neurons (Quarta et al., 2017). Therefore, we utilized the non-enzymatically active Rho specific inhibitor C3 toxin to address the role of RhoA in the activation of I_S1P_ in sensory neurons. Overnight pretreatment of DRG neuron cultures with C3 toxin (0.5 μg/ml) dramatically reduced I_S1P_ (Figures 4A,B, unpaired *t*-test, Control: 13.49 ± 3.28 pA/pF in *n* = 20 neurons, C3 toxin: 2.33 ± 0.55 pA/pF in *n* = 13 neurons, *p* = 0.0108), signifying that Rho signaling is critically involved in the activation of I_S1P_ in DRG neurons.

The Rho-associated protein kinase (ROCK) is a major downstream effector of Rho GTPase (Amano et al., 2010), we therefore tested whether ROCK is involved in the activation of I_S1P_ in sensory neurons using the ROCK specific inhibitor Y-27632 (10 μM, 30 min). No attenuation of I_S1P_ was observed in the presence of Y-27632 (Figures 4A,B, unpaired *t*-test, Control: 13.49 ± 3.28 pA/pF in *n* = 20 neurons, Y-27632: 9.02 ± 1.81 pA/pF in *n* = 14 neurons, *p* = 0.2966), suggesting that ROCK is unlikely contributing to I_S1P_ activation. Taken together, these results suggest that Rho but not its downstream effector ROCK is a critical component of I_S1P_ activation in sensory neurons.

We have previously reported that S1P induced Ca^2+^ influx in sensory neurons through chloride channel-dependent depolarization and concomitant activation of voltage-gated Ca^2+^ channels (Camprubí-Robles et al., 2013). To confirm the functional importance of Rho in the neuronal response to S1P, a Fura 2-based ratiometric calcium imaging technique was applied to measure S1P-induced Ca^2+^ transients after C3 toxin pretreatment. The proportion of neurons that displayed S1P-induced Ca^2+^ transients was significantly reduced from 59.9% (180/306 cells) in the control to 40.9% (103/273 cells) in the C3 toxin-treated neurons (Figure 4C, Fisher’s exact test, *p* < 0.001), corroborating the functional significance of Rho in the neuronal Ca^2+^ response to S1P in sensory neurons. In contrast, no significant differences were observed in the peak amplitude of S1P-induced Ca^2+^ transients between control and C3 toxin-treated groups (Figure 4D, Mann-Whiney U test, *n* = 103–180, *p* = 0.2234).

### S1P-Rho-Induced Cl^−^ Current Is Independent of S1P-Rho-Induced Potentiation of I_caps_ in Sensory Neurons

**Figure 5:**
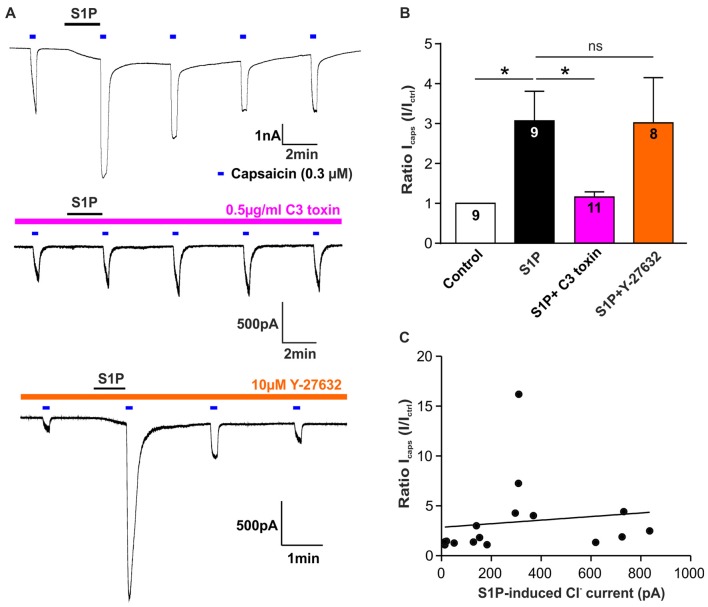
Rho but not ROCK is involved in S1P-induced I_caps_ potentiation in sensory neurons. **(A)** Typical voltage-clamp recording of current stimulated repetitively with capsaicin (0.3 μM) in DRG neurons treated without or with Rho inhibitor C3 toxin (0.5 μg/ml) or ROCK inhibitor Y-27632 (10 μM) at −60 mV holding potential. 1 μM S1P was applied between capsaicin stimulation. **(B)** Quantification of S1P-induced I_caps_ potentiation, which was normalized to the control I_caps_ peak amplitude (I_ctrl:_ I_caps_ before S1P application), in DRG neurons treated without or with C3 toxin or Y-27632. Exposure to S1P (1 μM, 1 min) significantly increased I_caps_ in DRG neurons (Unpaired *t*-test, control vs. S1P, *p < 0.05). C3 toxin (0.5 μg/ml, overnight) treatment significantly reduced S1P-induced potentiation of I_caps_ in DRG neurons (Unpaired *t*-test, S1P vs. S1P+C3 toxin, *p < 0.05). Y-27632 pretreatment (10 μM, 30 min) did not affect S1P-induced potentiation of I_caps_ in DRG neurons (Unpaired *t*-test, S1P vs. S1P+Y27632, *p* > 0.05). The number of recorded cells was noted in the bar. **(C)** Pearson correlation scatter plots showing that there was no linear correlation between S1P-induced Cl^−^ current and S1P-induced potentiation of I_caps_ (*n* = 16, *p* = 0.6293, *r*^2^ = 0.0171). I_S1P_ and I_caps_ were recorded on the same neuron.

We have demonstrated that S1P potentiated TRPV1-mediated currents induced by capsaicin (Mair et al., 2011; Langeslag et al., 2014), however, it is unclear whether the S1P-Rho signaling pathway is involved in this process. Thus, we explored if Rho signaling is also involved in S1P-mediated potentiation of capsaicin-evoked currents (I_caps_) in sensory neurons. As shown previously, exposure of S1P (1 μM, 1 min) significantly increased I_caps_ (capsaicin; 0.3 μM) in DRG neurons (Figures 5A,B, control vs. S1P, unpaired *t*-test, *n* = 9, *p* = 0.0133). This potentiation of I_caps_ by S1P was significantly reduced after overnight treatment of cultured DRG neurons with the Rho inhibitor C3 toxin (0.5 μg/ml, Figures 5A,B, fold increase, S1P: 3.07 ± 0.74 in *n* = 9, S1P+C3 toxin: 1.16 ± 0.13 in *n* = 11, unpaired *t*-test, *p* = 0.0120), suggesting that Rho is also involved in S1P-induced potentiation of I_caps_. Inhibition of ROCK activity with Y-27632 (10 μM) had no effect on S1P-induced I_caps_ facilitation (Figure 5B, fold increase: 3.02 ± 1.13 in S1P+Y-27632 group, unpaired *t*-test, *n* = 8–9, *p* = 0.9720). In summary, these data suggested that S1P activated I_S1P_ and induced potentiation of I_caps_ via signaling Rho but not ROCK in sensory neurons.

Based on these findings, it remains unknown whether the potentiating effect of S1P on I_caps_ was associated with I_S1P_ in sensory neurons. A correlation analysis of I_S1P_ amplitudes and S1P-induced ratio change of I_caps_ revealed that there was no linear correlation between S1P-induced potentiation of I_caps_ and the activation of I_S1P_ (Figure 5C, Pearson correlation coefficient, *p* = 0.6293). Despite the involvement of RhoA in both cellular effects, S1P-induced excitation through activation of a Cl^−^ current and the potentiating effect of S1P on TRPV1 function may be considered as independent processes in sensory neurons.

## Discussion

We have previously reported that the sphingolipid S1P excites DRG neurons through activation of a depolarizing chloride current (Camprubí-Robles et al., 2013). The present study revealed, for the first time to our knowledge, that chloride channels CLCN3 and CLCN5 mediated the activation of an excitatory current evoked by S1P in mouse sensory neurons. Furthermore, using electrophysiological recordings, ratiometric calcium imaging technique together with pharmacological inhibition approach, we showed that activation of I_S1P_ was dependent on Rho GTPase, but not ROCK. Additionally, we showed that, although utilizing similar cellular signaling components, I_S1P_ and S1P-induced potentiation of I_caps_ appear to represent independent events in sensory neurons.

Voltage-gated chloride channel CLCN family proteins are localized in plasma membrane and intracellular vesicles. In mammals, there exist nine different CLCN genes, which can be broadly grouped into two branches. One branch encodes plasma membrane Cl^−^ channels and includes the muscle-specific Cl^−^ channel CLCN1, the ubiquitously expressed CLCN2 and kidney-specific Cl^−^ channel isoforms CLCNKa and CLCNKb. The five CLCN channels (CLCN3–7) of the second branch are predominantly localized to endosomal and lysosomal membranes (Jentsch et al., 2005a,b). Using RNA sequencing followed by RT-PCR we identified four CLCN transcripts, CLCN3 to CLCN6 in sensory neurons.

Several studies have suggested that CLCN3, CLCN4 and CLCN5 are also located in the plasma membrane where they give rise to plasma membrane Cl^−^ currents (Steinmeyer et al., 1995; Friedrich et al., 1999; Wang et al., 2006; Cuddapah and Sontheimer, 2010; Reed et al., 2010). For example, CLCN3 is located at the plasma membrane of hippocampal neurons and contributes to a transmembrane Cl^−^ current in immature neurons (Wang et al., 2006). In cultured sensory neurons we have identified the chloride channels CLCN3 and CLCN5 that mediate an excitatory Cl^−^ current after S1P stimulation through an adenovirus-based gene silencing technique and whole-cell electrophysiological patch-clamp recordings.

The reduction of S1P-induced membrane depolarization after knockdown of CLCN3 and CLCN5 channels in mouse DRG neurons suggested that the membrane depolarization evoked by S1P is attributed to the activation of a Cl^−^ conductance. S1P may induce the activation of chloride channels CLCN3 and CLCN5, resulting in the Cl^−^ efflux in DRG neurons. Since the intracellular Cl^−^ concentration is high in primary afferent neurons and subsequently the Cl^−^ equilibrium potential is around −40 mV (Alvarez-Leefmans et al., 1988; Gilbert et al., 2007; Rocha-Gonzalez et al., 2008), the activation of CLCN3 and CLCN5 channels would result in Cl^−^ efflux causing depolarization of DRG neurons.

We and others showed previously in ion replacement experiments and with pharmacological inhibition that the S1P-induced current is carried by Cl^−^ in sensory neurons and neuroblastoma cells (Postma et al., 2001; Camprubí-Robles et al., 2013). Moreover, Postma et al. (1996, 2001) demonstrated that S1P-induced currents depolarized the membrane potential in the perforated patch-clamp configuration that keeps intracellular ionic conditions largely intact. In support of this, we also performed a small number of recordings in DRG sensory neurons with perforated patch-clamp technique and the S1P-induced current was indeed a depolarizing current also in sensory DRG neurons (data not shown). Thus, most probably S1P induces excitatory inward currents also under physiological conditions.

Previous studies have shown that a depolarizing Cl^−^ current and subsequent membrane depolarization can be evoked by various G-protein coupled receptors agonists such as S1P, LPA, thrombin and acetylcholine (Janssen and Sims, 1992; Postma et al., 1996, 2001), and the Cl^−^-dependent membrane depolarization has been reported in a broad ranges of cell types including both neuronal and nonneuronal cells (Kremer et al., 1989, 1992; Janssen and Sims, 1992; Postma et al., 2001; Camprubí-Robles et al., 2013). Cl^−^-dependent membrane depolarization may serve diverse physiological functions such as controlling membrane excitability in excitable cells and modulating Ca^2+^ signaling (Kremer et al., 1989; Postma et al., 2001; Camprubí-Robles et al., 2013). Since CLCN3 and CLCN5 are broadly expressed in tissues and cells (Steinmeyer et al., 1995; Jentsch et al., 2002; Fu et al., 2010), these two chloride channel (CLCN3 and CLCN5) may also play a role in the Cl^−^ current induced by other G-protein coupled receptors agonists such as LPA, thrombin and acetylcholine.

We observed that inhibition of Rho GTPase with its specific inhibitor C3 toxin greatly reduced the amplitude of S1P-induced Cl^−^ current and less DRG neurons responded to S1P with Ca^2+^ transients whose amplitude however was unchanged. This indicates that Rho GTPases may modulate Ca^2+^ transients through Cl^−^-dependent membrane depolarization. In the presence of the Rho inhibitor C3 toxin, the amplitude of S1P-induced Cl^−^ current was greatly reduced, which would reduce the level of membrane depolarization induced by S1P. The reduced membrane depolarization in the presence of C3 toxin may have less possibility of activating voltage-gated Ca^2+^ channels. As a result, less DRG neurons were responsive to S1P when imaging S1P-induced Ca^2+^ signal increases. Whenever the membrane is depolarized sufficiently by S1P to activate voltage-gated Ca^2+^ channel, the amount of Ca^2+^ influx might not be affected by Rho inhibitor. Subsequently, we would probably not be able to observe a significant difference in the amplitude of S1P-induced Ca^2+^ transients after C3 toxin treatment. In line with our study, RhoA is required for the activation of a Cl^−^ current by S1P in N1E-115 neuroblastoma cells (Ponsioen et al., 2009). This report together with our results, support a critical role for Rho protein for the activation of chloride currents by bioactive sphingolipids. Although Rho-kinase (ROCK), a serine/threonine kinase, is an important downstream effector protein of Rho GTPase (Amano et al., 2010), pharmacological inhibition of ROCK did not affect S1P-induced Cl^−^ current. This indicates that Rho *per se* or downstream effectors other than ROCK may act on chloride channels to activate the current. Since Rho activation is a critical process in S1P induced Cl^−^ current and RhoA can be activated by G_α13_ after receptor activation (Postma et al., 2001), it is most likely that activation of chloride currents occurred after S1P binding to the S1PR_3_ receptor expressed in sensory neurons (Camprubí-Robles et al., 2013).

At present, mechanistic insight into how Rho activation can be linked to the activation of a chloride channel such as CLCN3 and CLCN5 remains elusive. One possibility could be the directly or indirectly regulated trafficking of the channel towards the plasma membrane by Rho, a mechanism which has been demonstrated for CLIC4 and K_v_1.2 channels (Ponsioen et al., 2009; Stirling et al., 2009). On the other hand, chloride channel activity could be increased after, e.g., phosphorylation by Rho downstream signaling factors other than ROCK. Several phosphorylation sites have been identified in CLCN3 channel and its channel activity is dramatically potentiated after phosphorylation (Robinson et al., 2004; Cuddapah and Sontheimer, 2010; Ma et al., 2016). In addition, we have previously shown that p38/MAPK signal pathway is linked to the potentiation of TRPV1 activity by S1P (Langeslag et al., 2014), and in a recent report Rho can act as an upstream regulator of p38/MAPK (Shatanawi et al., 2011), thus p38/MAPK signaling may have a possibility of regulating the activation of Cl^−^ current induced by S1P. Further work is needed to elucidate molecular mechanisms underlying activation of CLCN channels by S1P.

Rho protein is not only a necessary factor for S1P-induced activation of a Cl^−^ current, but our study also showed that Rho signaling is required for S1P-induced potentiation of I_caps_ in sensory neurons, because inhibition of Rho by C3 toxin significantly reduced the potentiating effect of S1P on the TRPV1-mediated capsaicin response. Since the C3 toxin dampens the S1P-induced Cl^−^ current in capsaicin-responsive neurons, the question arises whether the S1P-induced potentiation of I_caps_ is dependent on or amplified by the S1P-induced Cl^−^ current. The signaling pathway that activates I_S1P_ and the signaling for S1P-induced potentiation of TRPV1 function both require Rho activation. However, the ROCK-independence of I_S1P_ activation and the absence of a significant correlation between I_S1P_ and S1P-induced potentiation of I_caps_ suggest that the signaling pathways may diverge after Rho activation. Unlike TMEM16a (ANO1), which can physically interact with TRPV1 and modulate each other’s activity (Takayama et al., 2015), there is no evidence showing a mutual functional interaction of TRPV1 with CLCN3 or CLCN5 in sensory neurons. However, CLCN3 and CLCN5 may modulate Ca^2+^-dependent ANO1 activity indirectly through activation of voltage-gated Ca^2+^ channels by S1P-induced membrane potential depolarization. Although the Rho GTPase effector ROCK has been suggested to be involved in heat shock-regulated TRPV1 activation (Iftinca et al., 2016), our data suggest that the S1P-evoked potentiation of I_caps_ like the activation of I_S1P_ in DRG neurons is independent of ROCK activity.

After tissue injury, high level of free S1P arises at local inflammation sites (Mitra et al., 2006; Hammad et al., 2008). S1P signaling has been known to be involved in many types of pain, e.g., inflammatory pain (Lai et al., 2008; Mair et al., 2011), postsurgical pain (Camprubí-Robles et al., 2013), cancer-induced bone pain (Grenald et al., 2017) and chemotherapy-induced neuropathic pain (Janes et al., 2014). However, to date, only few studies address the involvement of CLCN channels in pain initiation/modulation (Poët et al., 2006; Bali et al., 2013; Pang et al., 2016). The present study links the chloride channels CLCN3 and CLCN5 with the excitatory role of S1P on sensory neurons and for the first time positions CLCN5 channel, which has been well studied in Dent’s disease, an inherited renal disorder characterized by hyperphosphaturia, proteinuria, hypercalciuria and the development of kidney stones, which is often associated with mutations in the CLCN5 gene (Gunther et al., 2003), in the field of nociception and the pain pathway.

In conclusion, the present study demonstrates to our knowledge for the first time that chloride channels CLCN3 and CLCN5 are necessary components for S1P-induced chloride currents in sensory neurons. Furthermore, S1P induced the activation of I_S1P_ and potentiation of I_caps_ in a Rho-dependent manner, but S1P-induced potentiation of I_caps_ is independent of the S1P-induced Cl- current. Thus, novel mechanistic insight is provided into the regulation of sensory neuron function by bioactive sphingolipids.

## Author Contributions

YQ, ML and MK designed the research and wrote the manuscript. YQ, NM, MGL and MC-R performed the experiments. YQ, NM, KK, MGL and MC-R performed the analysis.

## Conflict of Interest Statement

The authors declare that the research was conducted in the absence of any commercial or financial relationships that could be construed as a potential conflict of interest.
