# Supplementary material for: Identification of Chloride Channels CLCN3 and CLCN5 Mediating the Excitatory Cl− Currents Activated by Sphingosine-1-Phosphate in Sensory Neurons
Source: Front Mol Neurosci. 2018 Feb 9;11:33. doi: 10.3389/fnmol.2018.00033 (PMC5811518; doi:10.3389/fnmol.2018.00033)
Supplement: Supplementary file 1 [file Data_Sheet_1.docx]

**Supplementary figure and figure legends**

**
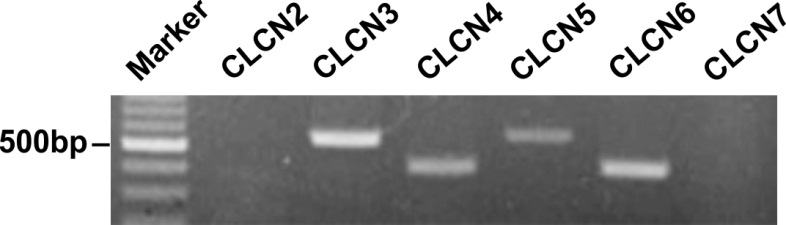
**

**Supplementary Figure 1.** **Expression of chloride channels in cultured sensory neurons.** Representative RT-PCR results for *Clcn2*, *Clcn3*, *Clcn4*, *Clcn5*, *Clcn6* and *Clcn7* mRNA in cultured DRG neurons. Marker: 100bp DNA ladder.

**
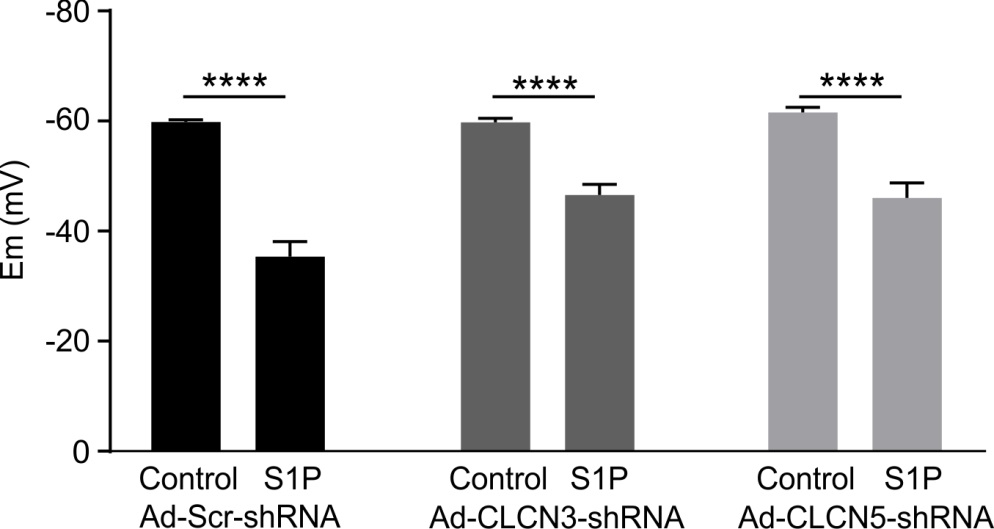
**

**Supplementary Figure 2. S1P induces a depolarization of membrane potential in cultured DRG neurons infected with adenovirus encoding scrambled shRNA, CLCN3 shRNA or CLCN5 shRNA.** Bar-chart showing application of S1P (1µM, 1 min) induced a significant change of membrane potential (E_m_) in DRG neurons with viral infection of Ad-Scr-shRNA (E_m_, Control (before S1P): -59.77 ± 0.38 mV, S1P: 35.28 ± 2.76 mV, unpaired t-test, n=29, **** *p*<0.0001), Ad-CLCN3-shRNA (E_m_, Control: -59.72 ± 0.71 mV, S1P: 46.49 ± 1.96 mV, unpaired t-test, n=35, **** *p*<0.0001) or Ad-CLCN5-shRNA (E_m_, Control: -61.48 ± 0.94 mV, S1P: 45.94 ± 2.78 mV, unpaired t-test, n=32, **** *p*<0.0001). Error bars indicate s.e.m.


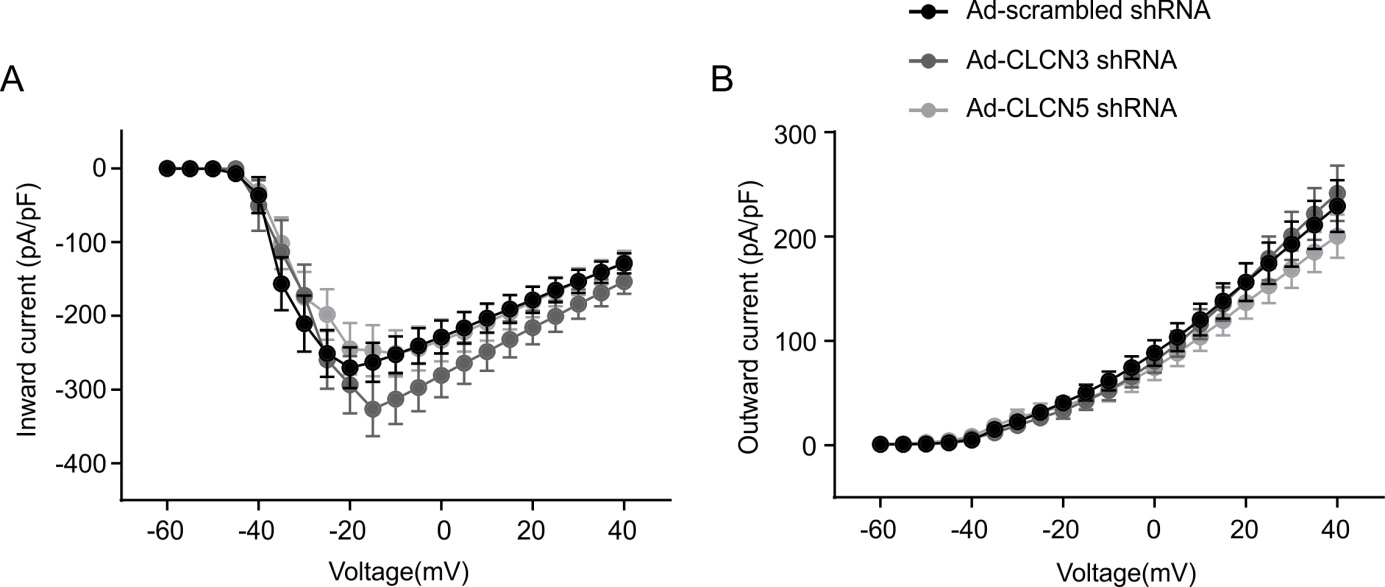


**Supplementary Figure 3. Effects of viral infection of Ad-CLCN3-shRNA or Ad-CLCN5-shRNA on voltage gated currents in cultured sensory neurons.** **A, B** Mean peak of whole-cell inward (**A**) and outward currents (**B**) measured at different test potentials from DRG neurons with infection of Ad-Scr-shRNA, Ad-CLCN3-shRNA or Ad-CLCN5-shRNA adenovirus. Neurons were depolarized from −65 to +40 mV in increments of 5mV. No significant change was seen in the voltage gated inward and outward current by adenoviral infection of Ad-CLCN3-shRNA or Ad-CLCN5-shRNA (Inward current: two-way repeated measures ANOVA, Ad-Scr-shRNA vs Ad-CLCN3-shRNA *p* > 0.05; Ad-Scr-shRNA vs Ad-CLCN5-shRNA *p* > 0.05; Outward current: two-way repeated measures ANOVA, Ad-Scr-shRNA vs Ad-CLCN3-shRNA  *p* > 0.05, Ad-Scr-shRNA vs Ad-CLCN5-shRNA *p* > 0.05). Error bars indicate s.e.m.


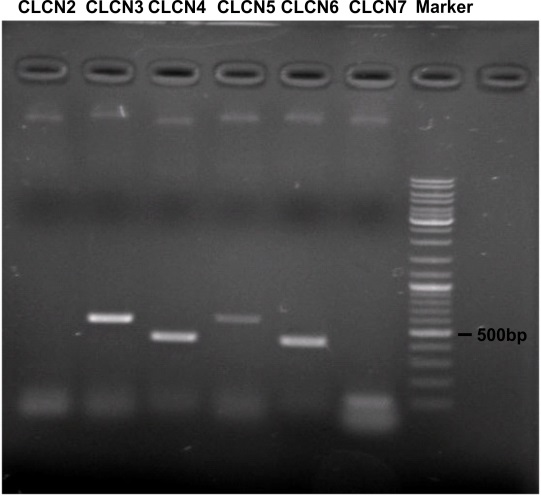


**Supplementary Figure 4. Original image for Figure 1A.**


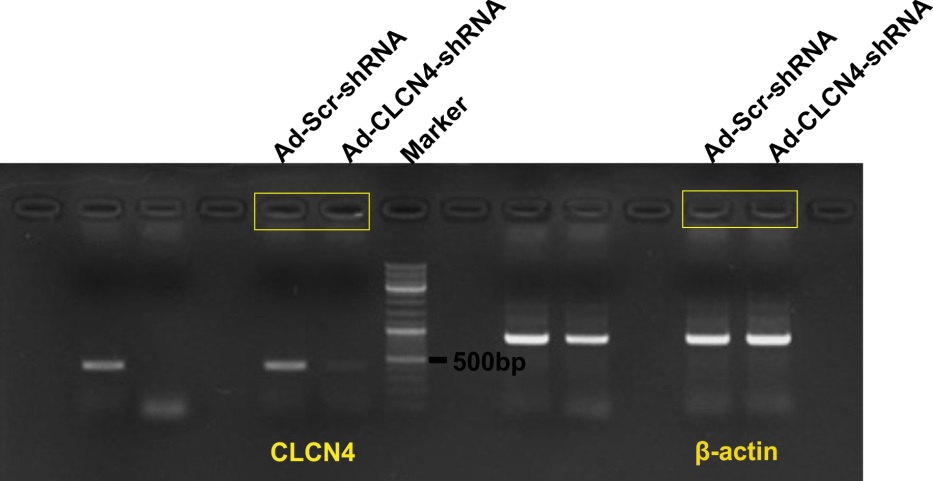


**Supplementary Figure 5. Original image for Figure 1B.**


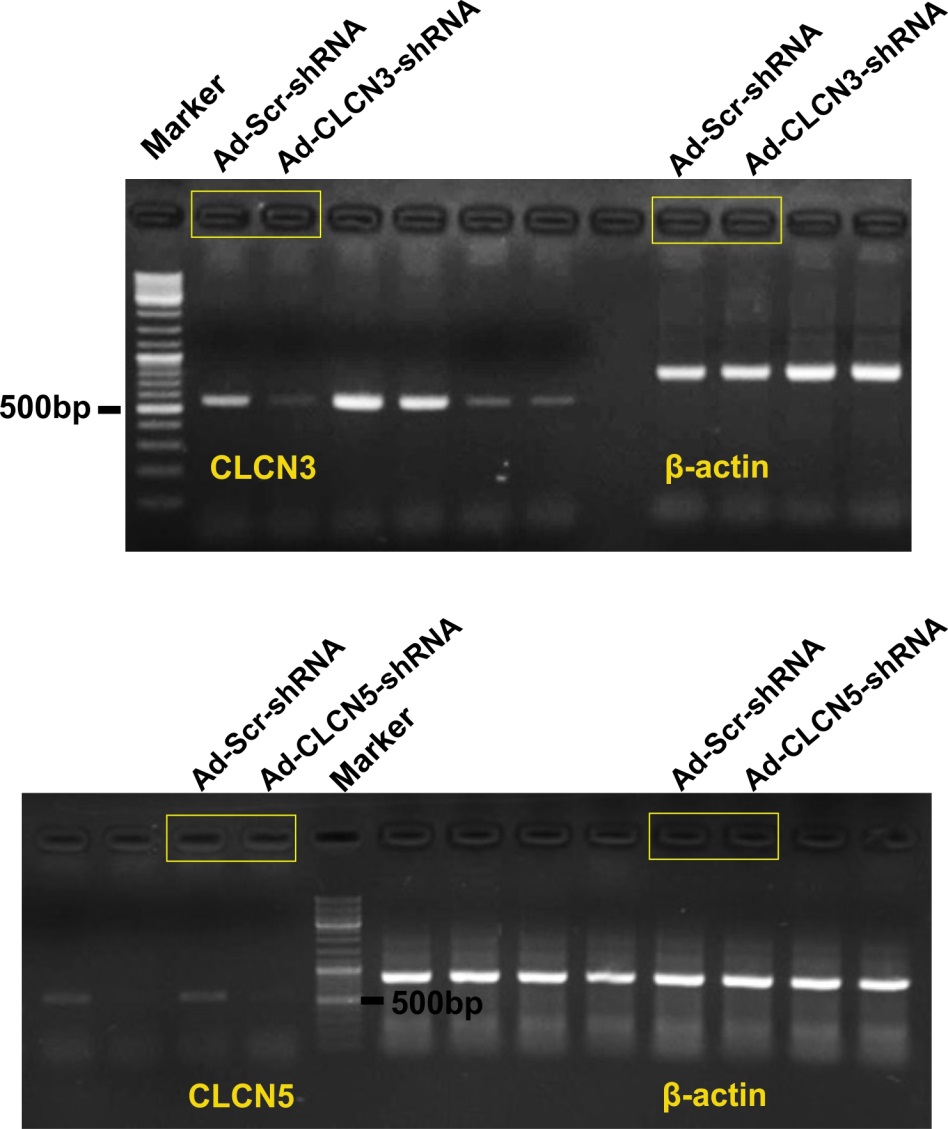


**Supplementary Figure 6. Original image for Figure 2A.**
